# Exploring Tai Chi in rheumatoid arthritis: a quantitative and qualitative study

**DOI:** 10.1186/1471-2474-11-43

**Published:** 2010-03-05

**Authors:** Till Uhlig, Camilla Fongen, Eldri Steen, Anne Christie, Sigrid Ødegård

**Affiliations:** 1 National Resource Center for Rehabilitation in Rheumatology, Diakonhjemmet Hospital, Oslo, Norway; 2 Department of Rheumatology, Diakonhjemmet Hospital, Oslo, Norway

## Abstract

**Background:**

Rheumatoid arthritis (RA) is a chronic, inflammatory and systemic disease which affects the musculoskeletal system. Exercise programmes are reported to improve physical functioning in patients with RA. Tai Chi is a traditional Chinese martial art which combines slow and gentle movements with mental focus. The purpose of this study was to study in which way Tai Chi group exercise impacted on disease activity, physical function, health status and experience in RA patients, applying quantitative and qualitative methods.

**Methods:**

Fifteen patients with RA (13 females, age 33-70 years) were recruited from a rheumatology department into a single group study. The patients were instructed in Tai Chi exercise twice weekly for 12 weeks. Assessments at baseline, 12 weeks, and 12 weeks follow-up were performed with a wide range of measures, including disease activity, self-reported health status, physical performance tests (Walking in Figure of Eight, Timed-Stands Test, and Shoulder Movement Impairment Scale). Qualitative data were obtained from a focus group interview conducted after completed intervention with taping and verbatim transcription. Review of the transcripts identified themes important to patients practicing Tai Chi.

**Results:**

Within the group, Tai Chi practice lead to improved lower-limb muscle function at the end of intervention and at 12 weeks follow-up. Qualitative analyses showed that patients experienced improved physical condition, confidence in moving, balance and less pain during exercise and in daily life. Other experience included stress reduction, increased body awareness, confidence in moving and indicated that Tai Chi was a feasible exercise modality in RA.

**Conclusions:**

Improved muscle function in lower limbs was also reflected when patient experiences with Tai Chi were studied in depth in this explorative study. The combination of qualitative and quantitative research methods shows that Tai Chi has beneficial effects on health not related to disease activity and standardised health status assessment, and may contribute to an understanding of how Tai Chi exerts its effects.

**Trial registration:**

NCT00522054

## Background

Rheumatoid arthritis (RA) is a chronic, inflammatory and systemic disease which predominantly affects the musculoskeletal system. The disease is often progressive and results in reduced physical function, pain, fatigue, and joint damage [1]. Exercise programmes are reported to improve physical functioning in patients with RA [2-5] by maintaining muscle strength, preserving joint mobility and maintaining flexibility, balance, endurance, and aerobic capacity [6]. Especially, low intensity exercises tailored to individual needs have been recommended for patients with RA [7].

Tai Chi is a traditional Chinese martial art that was developed in the 13th century, and combines slow and gentle movements with mental focus [8-10]. Intensity in Tai Chi is low and equivalent to walking 6 km/h, and gives a moderate increase in heart rate [11]. In the elderly Tai Chi improves agility and balance, postural control, lower extremity strength, physical function, prevent falls and increases flexibility [12-14]. In patients with arthritis improvements in pain and disability are observed [15], in osteoarthritis beneficial effects are shown for balance, abdominal strength, self-efficacy, quality of life and reduced pain and stiffness [16-18], in ankylosing spondylitis flexibility can be improved [19], while other studies examined Tai Chi in back pain [20] and osteoporosis [21].

In RA, Tai Chi leads to reduced disability and fatigue [22,23] and is considered safe [24]. The value of Tai Chi in RA is however still considered unproven [25], even though a Cochrane review on Tai Chi exercise concluded with positive effects on selected range of motion outcomes as well as increased level of participation and enjoyment of exercise for patients with RA [26]. Shortcomings of the reviewed studies for RA were lack of measurement of pain, balance or quality of life [27]. In a previously reported pilot study of Tai Chi in RA a short intervention period and physical functioning measures with floor effects limited conclusions, and further investigations of relevant outcomes in Tai Chi in RA were considered indicated [28].

Even though Tai Chi seems to be beneficial on a physiological and psychosocial level [11,29], we know little about the mechanism of benefit [29]. There seems to be a discrepancy between observed outcomes (e.g. falls) in spite of unchanged body functions (e.g. postural stability), which has been suggested mediated by increased confidence [30]. There is a lack of studies which explored patient experience during Tai Chi, examining whether quantitative physical or psychological outcomes correspond to how patients perceive Tai Chi.

The aim of this study was to examine how a standardised Tai Chi group exercise programme affected RA disease activity, physical function and health status, and how patients experienced Tai Chi during a focus group interview.

## Methods

### Participants

Fifteen patients with RA were recruited from the rheumatology department of Diakonhjemmet Hospital, Oslo, Norway, and the Oslo RA register [31]. Inclusion criteria were a diagnosis of RA according to the American College of Rheumatology (ACR) 1987 classification criteria [32], age 18-70 years, stable medical treatment, and no earlier experience with Tai Chi. Exclusion criteria were lack of ability to bear weight on the lower extremities, recent or ongoing disease flare, unstable heart condition, and participation in other physical exercise more than twice a week. The Regional Ethics Committee for Medical and Health Research approved the design of the study (reference number 587-03244). All subjects signed written informed consent before participating in the study.

### Intervention

Patients participated in a Tai Chi group exercise programme 'Twelve Movement Tai Chi for arthritis' [33]. This style applies small to large degrees of motion, knee flexion, straight and extended head and trunk, combined rotation of head, trunk and extremities and asymmetrical diagonal arm and leg movements [10]. The programme allows adjustment for movements to the functional level of the participant, and within the comfort zone either standing or sitting. The intervention was implemented as a group exercise twice weekly for 60 minutes over 12 weeks at the hospital gymnasium as in another study [34]. The leading instructor was supported by a co-instructor once a week. Both instructors were authorized in the 'Twelve Movement Tai Chi for arthritis' programme, and had extensive clinical experience in working with RA patients.

### Outcome measures

To explore any potential effects of Tai Chi on health a wide range of outcome variables was assessed one week prior to the start of the programme (T0), within one week after the end of intervention (T1), and at 12 weeks follow-up (T2). The assessments were performed around the same time of the day and by the same independent examiners who were not involved in the Tai Chi programme.

Demographic information with age, gender, disease duration, co-morbidity, education level and work situation were collected by self-report questionnaires.

A rheumatologist (SØ) assessed 28-swollen and 28-tender joint counts. Global assessment of disease activity was performed by patient and physician on 100 mm visual analogue scales. Disease Activity Score (DAS28) was calculated from erythrocyte sedimentation rate, swollen and tender joint counts, and patient assessment of global health [35].

Physical performance tests assessed balance, strength and endurance in lower-limb and shoulder function. Balance was measured with Walking in a Figure of Eight, counting numbers of missteps [36]. Strength and endurance were measured by Timed-Stands test, measuring in seconds the time used to rise up and sit down on a chair 10 times [37]. Shoulder function was measured with Assessments of Shoulder Movement Impairment Scale (SMIS, range10-60, 60 = best) which consists of 5 functional movements [38]. All physical performance tests were carried out by a physical therapist (AC).

Self-reported health status included Health Assessment Questionnaire (HAQ, range 0-3, 3 = worst) which is a widely applied measure for physical function [39], visual analogue scales (VAS, range 0-100 mm) for fatigue, muscle pain, and fear of falling [40], Short form Health Survey (SF-36, range 0-100, 100 = best) [41], and Arthritis Self-Efficacy Scales (ASES, range 10-100, 100 = best) [42]. ASES sub-scale for pain was not assessed at T0.

### Focus group interview

To weeks after the Tai Chi intervention all patients were invited for a focus group interview at the hospital to gain insight into how the participants perceived Tai Chi. The focus group interview is a well established research method where the participants, guided and encouraged by a moderator, reflect upon different aspects of specific questions designed by the researchers. The participants' experiences, options and concerns relating to specific topics are explored interactively [43,44]. One experienced moderator (ES) with knowledge of Tai Chi performed the interview lasting for one hour, applying a semi-structured interview guide (table 1). The interviewer encouraged participants to present opinions in their own words, and gave time to express differing opinions. When patients answered in general, they were encouraged them to be more specific and to give examples. For validation of statements and reflections the interviewer frequently asked *"Have I understood you correctly when I hear you say...?"*

**Table 1:** Interview guide used in the focus group

How was it for you to participate in Tai Chi?	
Do you in any way experience that Tai Chi	
had influence on your health?	
had influence on your daily life?	
lead to anything negative?	
How did you experience the group with respect to	
relations to other group members and to group leaders?	
structure (group size, duration of sessions)?	
If you were to recommend Tai Chi to others, what would you say?	
Would you like to add something?	

### Statistical analysis

Quantitative data were analysed using SPSS 12.0. Demographic variables were reported with median and range for continuous variables and as frequencies for categorical variables. The Wilcoxon signed rank test was used for within-group comparisons between T0-T1 and T0-T2. The level of significance was set to p < 0.05 and due to the exploratory nature of the study no corrections for multiple comparisons were performed.

### Content analysis

The qualitative data from the focus group were tape-recorded, transcribed verbatim and analysed using a step-by-step process of analysis according to Kvale [43]. A first analysis was made separately by two researchers, both physical therapists (CF, AC). Codes were identified to describe the content of the data, and categories where created. Thereafter the researchers met to examine their set of analyses to compare categories and identify overarching themes. The categories were then discussed until an agreement was reached. To ensure scientific rigour an experienced researcher (ES) validated whether the identified categories and overarching themes were in agreement with the raw data. Common traits and typical characteristics are presented in ordinary types, direct quotations in italic type.

## Results

### Quantitative assessment

Demographic variables of the participants in the Tai Chi group and focus group sub-sample are presented in table 2. Median attendance during the intervention was 20 (range 18-24) out of 24 sessions (83%). There were two dropouts in total, one patient undergoing RA related surgery during the intervention, and one patient who did not complete follow-up assessment. Ten patients reported practicing Tai Chi between classes, and eight reported continued Tai Chi practice at follow-up. No injuries were reported during exercise sessions and there were no changes in anti-rheumatic medication during the intervention or at follow-up.

**Table 2:** Baseline characteristics of Tai Chi participants and focus group subsample

	Tai Chi group (n = 13)	Focus group subsample (n = 9)
Gender female/male	11/2	8/1
Age (years)	57 (33-70)	57 (40-70)
Disease duration (years)	6 (1-45)	6 (1-45)
Co-morbidities (≥ 1)	6	4
Education		
≥ 12 years	10	8
< 12 years	3	1
Employment, full or part time	8	6
Disabled/retirement pension	5	3

Values are median (range) or number.

Disease activity and physical performance tests for all time points are given in table 3. Significant within-group improvements were found in number of swollen joints at end of intervention (p = 0.01) and at the follow-up (p = 0.02) and also for Timed-Stands test at the end of intervention (p = 0.01) and at the follow-up (p < 0.01). For disease activity DAS28 improvement (p = 0.04) was found at the end of intervention but not at follow-up. No changes were seen for the results of balance test and SMIS test.

**Table 3:** Disease activity and physical performance tests measured at baseline (T0), end of intervention (T1), and 12 weeks follow-up (T2) (n = 13)

	T0	T1	p-value (T0-T1)	T2	p-value (T0-T2)
*Disease activity*					
DAS28	4.7 (2.2-6.7)	4.1 (1.1 - 6.0)	0.04	4.7 (0.8-6.5)	0.24
No. swollen joints (0-28)	11 (2 - 20)	7 (0 - 17)	0.01	5 (0 - 14)	0.02
No. tender joints (0-28)	8 (1 - 29)	4 (0 - 22)	0.22	9 (0 - 20)	0.40
Patient global assessment (0-100)	31 (1 - 51)	21 (1 - 45)	0.67	43 (6 - 61)	0.39
Doctor global assessment (0-100)	32 (19 - 72)	26 (3 -57)	0.08	34 (0 - 55)	0.42
*Physical performance test*					
Figure-of-Eight balance	2.5 (0 - 12)	4 (0-11)	0.24	2.5 (0 - 10)	0.88
Timed-Stands Test (sec)	23 (13 - 34)	15 (10 - 26)	0.01	16 (10 - 28)	< 0.01
Shoulder Movement Impact Scale (0-60)	59 (46 - 60)	59 (46 - 60)	0.10	59 (41 - 60)	0.08
Pain during SMIS (0-100)	8 (0 - 34)	4 (0 - 22)	0.06	10 (0 -19)	0.70

Values are median (range)

DAS28 = Disease activity score

Table 4 presents health status measured at baseline, end of intervention and 12 weeks follow-up. Statistically significant improvement was only found for the SF-36 social functioning scale (p = 0.03) between baseline and 12 weeks, but not at follow-up and neither for any other standardised self-reported variable of health status.

**Table 4:** Health status measured at baseline (T0), end of intervention (T1), and 12 weeks follow-up (T2) (n = 13)

	T0	T1	p-value (T0-T1)	T2	p-value (T0-T2)
HAQ (0-3)	0.5 (0.0-1.5)	0.5 (0.0-1.2)	0.72	0.5 (0.0-1.5)	0.34
Fatigue (0-100)	27 (5 - 89)	28 (3 - 81)	0.55	25 (6 - 74)	0.70
Muscle pain (0-100)	30 (1 - 60)	25 (2 - 69)	0.92	23 (2 - 61)	0.88
Fear of falling (0-100)	14 (0 -76)	21 (2 - 71)	0.75	33 (10-95)	0.13
SF-36 (0-100)					
Vitality	45 (15 - 80)	55 (35 - 70)	0.26	50 (25 - 70)	0.59
Pain	51 (0 - 84)	62 (41 - 84)	0.05	42 (22 - 100)	0.17
General health	52 (35 - 82)	50 (32 - 87)	0.96	47 (35 - 97)	0.92
Mental health	80 (40 - 92)	76 (36 - 92)	0.68	76 (56 - 92)	0.59
Physical functioning	75 (25 - 95)	70 (30 - 95)	0.72	75 (25 - 95)	0.91
Social functioning	75 (12 - 100)	75 (25 - 100)	0.03	62 (12 - 100)	0.22
Role emotional	100 (0 - 100)	100 (33 - 100)	0.23	67 (0 - 100)	0.86
Role physical	50 (0 - 100)	75 (0 - 100)	0.19	25 (0 - 100)	0.91
Self-efficacy (10-100)					
Pain	NA	70 (18 - 100)	NA	62 (18 - 86)	0.45*
Function	89 (54 - 100)	88 (28 - 99)	0.40	90 (61 - 100)	0.22
Symptoms	75 (35 - 90)	75 (42 - 100)	0.45	78 (57-97)	0.13

Values are median (range).

*T1 versus T2

NA = not assessed

HAQ = Health Assessment Questionnaire

SF-36 = Short Form Health Survey 36

### Focus group interview

Nine of the 13 patients completing follow-up attended the focus group interview (table 2). Analyses identified six overarching themes: experienced effects of Tai Chi exercise, daily usefulness Tai Chi, desire for further Tai Chi practice, the group process, instructors, and practical implementation.

#### Experienced effects of Tai Chi exercise

Patients expressed positive effects experienced during Tai Chi, reporting less pain in shoulders, knees, ankles, and headache, and improved peripheral blood circulation, posture, balance, coordination and vitality.

"When I started with Tai Chi my shoulders where very bad and now they are fine"

[Female, 70 years].

*"After the Tai Chi class I was full of energy and ready to face my life" *[Female, 59 years].

The patients experienced an increased body awareness related to breathing, difficult movements and generally more contact with their body. They also described an increased relaxation of body and mind while doing Tai Chi.

*"The relaxation I experienced while doing Tai Chi, is both physical and mental" *[Female, 57 years].

One patient reported increased pain at the start of the intervention, followed by a reduction to a lower level than before participation.

#### Daily usefulness of Tai Chi

Patients expressed improvement in walking by being able to walk longer, faster and with a better balance, stability, coordination and less pain.

"*After I started with Tai Chi I'm able to use my joints without so much pain or without an increase of the pain after walking" *[Female, 65 years].

The patients described benefits also for other physical activities. They also expressed more confidence in difficult movements, by experiencing an increased mental awareness.

*"Due to increased awareness I can take a deep breath and be more present in a difficult movement, like picking up something from the floor, this makes the movement easier*" [Female, 45 years].

*"I'm now walking much more and dare to use my knees" *[Female, 40 years]

Some used Tai Chi as a method for stress reduction both at home and at work.

*"I stop in rather stressful situations at the office and do Tai Chi which helps me to become more relaxed" *[Female, 56 years].

#### Desire for future Tai Chi exercise

Tai Chi was experienced as a suitable form of exercise in RA worth while to continue practicing.

*"There is no doubt that we wish to continue with Tai Chi" *[Male, 60 years].

#### The group process, instructors and practical implementation

The effect of participating in a group contributed to well being and encouraged doing physical exercises regularly.

*"It was very positive to be able to participate in a physical exercise group and still feel well" *[Female, 57 years].

Competence with the instructors in Tai Chi, didactic skills and adjustment to the level of group members were important.

*"I felt that the leader had good theoretical knowledge, and was able to convey it in a practical way, so I felt that Tai Chi is working for me'*" [Female, 65 years].

The patients had a positive experience of learning the basics of the different movements before learning the details. It was valuable to start every class by repeating the training from the previous session.

*"For me it was right to start learning the basic of the movements, before you start working with details" *[Female, 48 years].

The use of Tai Chi music helped getting into the mood for exercise, and made it easier to combine breathing with movements.

## Discussion

In this study we chose a broad and explorative approach to study how Tai Chi could affect body functions, health outcomes and how patients with RA experienced Tai Chi. We found that participating in a 12-week Tai Chi group programme improved lower-limb muscle strength and endurance as well as swollen joints at 12 weeks follow-up after the programme. These quantitative physical improvements were reflected by results from the focus group, thus increasing the validity of the findings. Qualitative analyses also identified themes such as desire for further Tai Chi practice, and practical implementation which indicate that Tai Chi can be implemented into daily life of individuals with RA.

Muscle strength and endurance are important aspects of physical functioning for patients with RA. Observed improvement in muscle function of the lower limbs as measured by the Timed-Stands test in this study is supported by patient experience of improved walking ability and more confidence while moving. In patients with RA, Timed-Stands test is highly influenced by disease related factors which affect lower-limb function, such as pain, tender joints, inflammation and weakness [37]. In our study contributing factors to improvement could be increased strength due to exercising with bended knees and pain reduction, as the participants described increased stability and reduced pain in knee and ankle joints.

In a systematic review, no statistically significant difference between a Tai Chi group and a control group regarding number of swollen joints was reported [26]. Disease activity in this study was not improved even though swollen joints are integrated into calculation of DAS28. Taken together with potential of bias in our single sample study and with lack of similar results in the qualitative approach we find no convincing evidence that that Tai Chi should decrease disease activity in RA, consistent with our clinical understanding of RA [45].

In other reports Tai Chi has lead to reduced pain in patient with osteoarthritis [17,18], but not in RA [22,24]. In the focus group interview in our study patients reported reduced pain in joints and more vitality, but consistent pain improvement or improvement in other health status measures was not found in the quantitative assessments. Thus, patient experience in our study and popularity of Tai Chi in general indicate positive health effects which are not necessarily picked up by current instruments.

The value of regular physical activity is well documented, both in the management of RA and for secondary disease prevention [4,5,46,47]. Patients with RA are less physically active than the general population. Physical activity for them needs to be sustainable and enjoyable [48]. Results from the focus group interview suggest that participating in Tai Chi group exercise contributed to increased confidence in moving and group members supported each other to be physically active and enhanced enjoyment during exercise. Given good compliance, lack of injuries, experience of enhanced physical activity as well as continued practice of Tai Chi during the follow-up indicate that patients with RA increase their level of physical activity when practicing Tai Chi.

Our approach with qualitative research identified additional areas of health benefit not observed in the quantitative assessments. One reason for this might be that two of the physical performance test, SMIS and Walking in a Figure-of-Eight, showed a considerable floor effect. Effects of Tai Chi on stress reduction [11] and improved balance [13,17] have been demonstrated in other studies, but not in patients with RA. It is a conundrum that Tai Chi exercise may have benefits that are not reflected by functional assessment, for example reduced falls in the light of unchanged postural stability [30]. Such findings lead to a hypothesis that psychosocial factors (e.g. increased confidence) may alter an outcome. In that light our findings of increased confidence in moving and body awareness are supportive, but have otherwise to our knowledge not been addressed before. Further research needs to be done on Tai Chi to validate the results found in this study and, in particular, to study in patients with musculoskeletal disease body control in movement, muscle strength and endurance, as well as aspects of body awareness.

A major limitation in this pilot study is the exploratory single group design with a small number of subjects and the lack of a control group, which make it possible that other factors than the nature of the intervention itself could explain observed changes in outcomes. This was as expected and the constitution of the group was heterogeneous, enhancing exploration of Tai Chi, but also limiting the ability to discover any clear conclusions on the studied phenomenon. Strengths of the study were recruitment of participants from a rheumatology out patient department among correctly classified patients under medical attention for their disease, independent examiners who performed all assessments, an experienced interviewer for the focus group, and competence in analysing quantitative outcomes. Further, to enable thorough exploration, we applied a wide range of measures for assessment of disease activity, performance and health status.

In this study both quantitative and qualitative research methods indicated positive physical health effects of Tai Chi. Psychosocial health effects were most consistently seen in the qualitative analyses. Further research on psychosocial factors with focus on patient experience and patient perspective [49] will contribute to a broader understanding of Tai Chi.

## Conclusions

In the present study quantitative and qualitative research indicates improved health outcome of Tai Chi mainly related to muscle strength and endurance. The focus group interview indicated additional categories important in Tai Chi which point to the importance of psychosocial factors not identified by standard assessment of patients. The combination of both quantitative and qualitative research methods in this study allowed for a broader understanding of health effects exerted by Tai Chi exercise.

## Competing interests

The authors declare that they have no competing interests.

## Authors' contributions

TU and CF initiated the study, design was undertaken by TU, CF and ES. Funding was supplied by the institution. CF managed the project, CF and TU undertook the recruitment and treatment. Assessments were performed by SØ, AC and ES. CF and TU undertook statistical analysis. CF and TU wrote the draft manuscript. SØ, ES, and AC revised the manuscript and with CF, and TU gave approval of the final version submitted. TU takes responsibility for the accuracy and honesty of the report and for the ethical aspects of the study.

## Pre-publication history

The pre-publication history for this paper can be accessed here:

http://www.biomedcentral.com/1471-2474/11/43/prepub
